# PaCE Yourself: Impact of COVID-19 on Patient-Centered Care Experience

**DOI:** 10.3390/pharmacy9010043

**Published:** 2021-02-18

**Authors:** Kristen Wilhite, Mikael Jones, Clark Kebodeaux

**Affiliations:** College of Pharmacy, University of Kentucky, Lexington, KY 40508, USA; Mikael.jones@uky.edu (M.J.); Clark.kebodeaux@uky.edu (C.K.)

**Keywords:** COVID-19, student pharmacists, online, simulation, teaching and learning, assessment

## Abstract

(1) Background: The outbreak of the novel coronavirus, COVID-19, forced colleges of pharmacy to implement new online learning methodologies to ensure that students could complete required courses. This transition was especially acute for laboratory simulation courses that require students to practice professional skills. This study aims to compare student assessment performance within a simulation-based laboratory course for students who completed the module prior to and after the online transition. (2) Methods: This study was a retrospective cohort comparison of student outcome performance with two distinct content delivery methods. Students were organized into two tracks at the beginning of the semester to determine the order of the simulation module. The online learning transition occurred in-between the delivery of the same module, which allowed comparison of online versus in-person content delivery with consistent assessment. Remediation rates on each assessment were compared using chi-squared tests. (3) Results: Student pharmacists across the first and second professional year performed similarly despite in-person or online course formats, with no significant differences in remediation rates. (4) Conclusions: Pharmacy course content, including laboratory-based simulation activity, may produce similar assessment performance when using online content delivery. Further research into hybrid or mixed-delivery models may enhance learning without affecting assessment performance.

## 1. Introduction

The novel coronavirus, COVID-19, has impacted more than 4000 colleges and 25 million students in the United States due to campus closings, and required many courses to shift to an online format [1]. While most students already utilize some form of online learning, the rapid transition from in-person to online learning format during what was the middle of a semester for most required educators to utilize innovative and unfamiliar technology to ensure student success [2]. The COVID-19 pandemic provides a unique opportunity for educational institutions to explore online learning, which closely follows developing trends in higher education [3]. While there are challenges with implementing online learning, most students are receptive and are still able to successfully learn from this style of education [2,3,4,5].

Despite the rapid online transition of course instruction, online learning is not a novel concept. The utility of online learning has been studied for nearly 20 years, including in the United States and globally, with evidence finding that it is at least as effective as in-person learning [6,7,8]. Cook D.A. et al. performed a meta-analysis that reviewed online learning in health professions and found that overall satisfaction, knowledge, and skills outcomes were as effective as traditional (in-person) interventions [8]. While students still tend to prefer in-person learning, there is evidence that as long as the course instructor has good communication, students can be equally satisfied with online courses compared to in-person [9,10,11]. In fact, online learning may provide more opportunities for collaborative learning, and allowing educators to hold more of a “facilitator” role when teaching, rather than simply lecturing to students [12].

Transitions towards blended learning, where education is split between online and in-person learning, is where healthcare professional education has been trending [13]. Most pharmacy schools are already equipped to utilize online and technology-based learning resources, so transitioning from in-person to online learning may be more seamless than expected [14,15]. Although pharmacy education is becoming more technologically based each year, some student pharmacists still have resistance to the idea of less in-class learning [16]. While a fully online education may not appeal to all students, Ruehter V. et al., along with Crouch M.A.’s studies, have shown that blended learning and supplementary online education still can be beneficial [17,18].

Implementing online learning into lab-based courses that typically require hands-on practice may prove more of a challenge compared to a standard lecture-based course. Some skills can only be taught in-person, but others, such as patient counseling and even certain skill activities, can be effectively taught through recorded or live online lectures [19,20,21]. Given the transition that occurred in March of 2020 in the United States due to COVID-19, the aim for this study is to compare the success in assessment between student pharmacists that were taught in-person versus online based on remediation rates on common assessments.

## 2. Materials and Methods

Patient-Centered Care Experience (PaCE) is a six-semester course sequence at the University of Kentucky College of Pharmacy. The courses are designed to assist students in developing the knowledge, skills, and attitudes needed to fulfill responsibilities necessary to provide patient-centered care and manage the medication use system. All competencies that are assessed during PaCE are based on the Center for the Advancement of Pharmacy Education (CAPE) and Accreditation Council for Pharmacy Education (ACPE) accreditation requirements [22,23]. The PaCE curriculum is broken down into three components: community service learning, experiential education, and simulation. Community service learning allows students to serve others, as well as engage with a team of peers and a mentor. During a student’s longitudinal experiential education, they are able to practice pharmacy in action and put their knowledge into real-world context. The simulation component of PaCE is intended to introduce students to foundational concepts, provide practice opportunities in a safe, controlled environment, and assess their skills on key module competencies.

PaCE simulation consists of institutional, patient care, and ambulatory care modules. The institutional module focuses on inpatient pharmacy tasks, such as sterile compounding. The patient care module encompasses a variety of topics ranging from patient counseling to training for clinical protocols. Lastly, the ambulatory care module typically covers non-sterile compounding along with outpatient community-based counseling skills and smoking cessation counseling. Although this study is specifically assessing the ambulatory care module, each lab module is set up similarly, in that they span over 4 weeks, with the final week including an assessment of key module competencies. If a student fails to meet competency by scoring less than an 80% on a specific section of the initial assessment, they will have two additional attempts to remediate the activity and demonstrate mastery of the module to pass the course. PaCE competency assessments are designed to provide students with personal and professional feedback based on their performance, with the opportunity to review and further practice the material under the guidance of a professor if initial competency is not met to ensure full mastery of the material. Along with competency assessments, students are also given professionalism scores throughout each semester, with which points are deducted based on timeliness of attendance and submission of assignments. Online students were held to the same professionalism standards as students that completed the module via an in-class format. 

Unique to PaCE is a layered-learning approach that requires students to learn in a near-peer environment. The near-peer environment allows for students that are further into their pharmacy education, such as PY2 and PY3 students, to teach students, such as PY1s, creating a layered model of learning. Each section of simulation, regardless of content area, contains students from all three years of the didactic curriculum. Sections are evenly distributed between professional year 1, 2, and 3 (PY1–3) students. This provides students with the opportunity to experience, learn, and provide feedback based on the level of individual learner around a provided set of learning outcomes. However, due to class schedules and available laboratory space, students are unable to complete each section of simulation simultaneously. Course progression is achieved by dividing up the PY1, PY2, and PY3 classes in half into two tracks: track A and track B. An assigned track determines the order of simulation modules a student completes.

In March 2020, the University of Kentucky, including the College of Pharmacy, moved all instruction to remote learning utilizing online teaching methods due to the COVID-19 pandemic. Given the structure of PaCE, track A (in-person) had already completed the ambulatory care module and first remediation, while track B (on-line) would be required to complete the same ambulatory care module in a virtual format.

Great care and effort were taken to maintain the fidelity of the ambulatory care module despite the online nature of the course. Each simulation section schedule was maintained and near-peer activities were conducted via recorded Zoom™ meetings. Each section required a Zoom™ meeting for PY1, PY2, and PY3 students, and students moved in and out of regularly scheduled sections as required by near-peer activities. Examples, including peer-evaluation of simulated smoking cessation encounters between PY1 (patient), PY2 (pharmacist), and PY3 (preceptor), were transitioned from in-person recorded encounters to online recorded encounters via Zoom™.

Assessment techniques between track A and track B were kept identical when possible to accurately measure competency. Most assessments had a digital component when performed in-person; learning objectives and qualitative differences in assessments are detailed in Table 1. The consistency in assessment between track A and track B allowed for performance comparison in the delivery of content. Additional time to submit the assignment was allotted for certain online assessments to account for any potential technical difficulties or delays. 

A quantitative assessment was used to evaluate the primary and secondary outcomes of interest. The primary outcome was the number of students that had to remediate a given exam portion in the PY1 and PY2 classes. The secondary outcome was the different professionalism scores between track A (in-person) and track B (online) students. The number of students that passed or failed a given assessment portion in both the online and in-person tracks were downloaded and stored in Microsoft Excel spreadsheets and retrospectively evaluated. Descriptive statistics were reported as the total number of students for each exam outcome and differentiated into track A and track B students. A chi-squared analysis was used to compare the two tracks, with a *p* value < 0.05 considered statistically significant.

## 3. Results

Between track A and track B, a total of 264 PY1 and PY2 students completed PaCE in spring 2020. PY3 students completed a longitudinal protocol implementation project and were not included in this analysis. All students eventually met competency for the ambulatory care component of PaCE simulation. A total of 307 second remediation attempts between both track A and track B were completed online.

Table 2 summarizes the remediation rates after the initial assessments in both track A (in-person) and track B (online). There was no difference in remediation rates after the initial set of assessments (Table 2). The second assessment attempt was completed online by both track A and track B. There was also no difference in remediation rates after the second attempt, as shown in Table 3.

The average professionalism score for track A students in both the PY1 and PY2 courses was 98.03%, compared to track B students with an average professionalism score of 97.69%. Only seven students in track A and eight students in track B had professionalism scores below 90%. Based on the similar average scores, timeliness and punctuality did not seem to differ between each track, and were likely not factors that played a role in the ability of students to meet assessment competencies.

## 4. Discussion

There was no significant difference in the remediation rates between in-person and online tracks between all assessments. Each track completed the same pre-assessment training and education, and the assessments were intended to be as similar as possible, aside from delivery. The lack of difference between the remediation rates indicates that online simulation learning and subsequent assessments were at least as effective as in-person learning and assessments.

The total points, number of exams, and content of the exams was kept consistent between the two tracks. The online track (track B) received a 20% time increase compared to the in-person track (12 min vs. 10 min) in order to accommodate for potential online submission issues. Due to the limitations of the remote exam, track B was also unable to physically compound products, but all learning objectives were maintained. The lack of physical compounding may be seen as an advantage or disadvantage to the online students. Although it did not seem to significantly impact remediation rates, these students most likely are not at the same competency level for non-sterile compounding compared to their peers that were able to learn hands-on compounding techniques. The lack of on-hands training may be a dramatic limitation that will not be revealed until later in the student’s education. This study was not able to assess long-term impacts, which may be a source of interest in future studies.

While there was technically no significant difference between the remediation rates, the track A group ( in-person) did have more students that failed on their second and third attempts compared to the track B group. One explanation for this may be that the track A students did not have prior experience with the online assessment set-up, causing more of them to not meet competency. The track B students may have had a slight advantage, given their initial assessment was administered using the online format.

There are limitations to the study results. The most significant limitation is the potential for proctored versus un-proctored exams in the in-person and online assessments, respectively. While students were reminded to follow the Honor Code within the College of Pharmacy, it is expected that some students may have utilized outside resources and peers while taking an online un-proctored exam. This may have resulted in the online track (B) having had lower remediation rates than they would have if given a proctored exam.

Another limitation of this study is the lack of comparison between actual percentage scores, rather than a basic pass/fail score. It may be that both tracks had similar pass/fail rates, but there may have been significantly different percentage scores that were not accounted for due to anything above or below an 80% being registered as a simple pass or fail, respectively. However, since the course is competency-based, all students met the requirement for progression and experiential learning. Along with the lack of percentage score assessment, student demographics, such as age, internet access, GPA, and previous online usage, were also not reviewed.

Although students will eventually be able to attend all courses in-person once the COVID-19 pandemic has resolved, online education will continue to be a significant aspect of many pharmacy curriculums. The online format allows students to have easy access to their courses, along with providing an environment where students are able to work at their own pace, removing potential distractions from classmates. It is important to consider the ongoing utility of online learning and ensure that students are still able to succeed with this form of instruction. 

Along with our study, other studies have reported similar findings regarding student success with an online learning format. As mentioned previously, even before the COVID-19 pandemic pushed education online, studies showed that students were succeeding when learning laboratory-based material online. In the study completed by Reuter R., where students completed a laboratory class online or on-campus, online students even improved their grade percentage more than those learning on-campus [6]. On the other hand, a study that analyzed pharmacy students’ performance in-person versus those receiving instruction through a video conference found that students learning via video conference had lower overall course grades and confidence in their success [24].

Much of the primary literature regarding online versus in-person learning tends to show contradictory results, which leaves plenty of room for further advances in research regarding this subject. Potential future studies could assess long-term impacts of online vs. in-person learning on the success of students in the following semesters, as well as during experiential education. As online learning is increasingly incorporated into curriculums, the ability of students to retain information provided virtually will certainly be of interest. Studies can also assess the students’ overall satisfaction with in-person laboratory skill learning compared to online learning, and further evaluate differences between students that prefer online versus in-person learning. Analyzing the ability of students to complete simulation-based laboratory learning via different software programs and video lectures and implement that knowledge in practice is also an area where further research is needed.

## 5. Conclusions

Due to the COVID-19 pandemic, half of the pharmacy students completing an ambulatory care lab course were made to complete the course and its subsequent exams online. The other half of the pharmacy students were able to complete the lab course in-person, before on-site access was restricted. Based on our results, it does not appear that remediation rates were impacted by online vs. in-person learning in the simulation-based setting. Educators may be able to incorporate more online learning for their students if needed, particularly when utilizing simulation-based activities, and feel confident that students are still acquiring competency when utilizing this model.

## Figures and Tables

**Table 1:** Ambulatory care module assessments.

Assessment	Learning Objectives	Assessment Technique In-Person (Time Limit)	Changes to Assessment Technique when Administered Online (Time Limit)
PY1 Checking Station	Review a prescription to determine if it is valid, complete, indicated, and dosed appropriately for the patient’s indication	Checking Station: processed prescription bottle, label, and handwritten prescriptions (10 min)	Digital Picture of Checking Station: processed prescription bottle, label, and handwritten prescriptions (12 min)
PY1 MyDispense	Identify the presence of a technical problem with a prescription and resolve the problem appropriately without introducing new errors	Two assessment exercises: one self-care and one prescription (20 min)	No change (20 min)
PY1 Aliquot	Preform an aliquot calculation (calculator use permitted) in order to accurately prepare a non-sterile product using available supplies	Calculation-based worksheet to accurately assess calculation competency (10 min)	No change (12 min)
PY1 Compounding	Use a formulation record to correctly prepare a non-sterile solution or suspension using appropriate technique and equipment	ExamSoft-based 10-question assessment based on calculations needed with new prescription and master formulation compounding record (MFCR) (20 min)	No change (20 min)
PY1 Geometric Dilution	Prepare a non-sterile product using a geometric dilution technique	Students were observed via an Observed Structured Clinical Examination (OSCE) checklist to confirm appropriate technique (10 min)	Students were assigned to one of four videos and had to confirm appropriate or inappropriate technique (15 min)
PY2 Smoking Cessation	Complete a standard patient encounter using the 5 As and smoking cessation protocols	Students completed an OSCE assessment with a standardized patient in-person (8 min)	Students completed an OSCE assessment with a standardized patient via Zoom (8 min)
PY2 MyDispense	Identify the presence of a technical problem with a prescription and resolve the problem appropriately without introducing new errors	Two assessment exercises: one self-care and one validation (20 min)	No change (20 min)
PY2 Compounding	Use a formulation record to correctly prepare a non-sterile solution or suspension using appropriate technique and equipment	Students approved or rejected a completed compound based on a standard quality control checklist (10 min)	No change (20 min)

**Table 2:** Student remediation rate after first attempt.

	Total Student Attempts	Total Students Not Meeting Competency (%)	Total Track A Attempts	Track A (%) In-Person	Total Track B Attempts	Track B (%) Online	*p* Value *
PY1 Checking Station	134	40 (29.9)	68	25 (37.6)	66	15 (22.7)	0.08
PY1 MyDispense	134	26 (19.4)	68	17 (25)	66	9 (13.6)	0.10
PY1 Aliquot	134	66 (49.3)	68	34 (50)	66	32 (48.5)	0.86
PY1 Compounding	134	37 (27.6)	68	21 (30.8)	66	16 (24.2)	0.39
PY1 Geometric Dilution	134	25 (18.7)	68	13 (19.1)	66	12 (18.2)	0.89
PY2 Smoking Cessation	130	17 (13.1)	65	10 (15.4)	65	7 (10.8)	0.44
PY2 MyDispense	130	28 (21.5)	65	15 (23.1)	65	13 (20)	0.67
PY2 Compounding	130	68 (52.3)	65	38 (58.5)	65	30 (46.2)	0.16

* Chi-squared analysis.

**Table 3:** Student remediation rate after second attempt.

	Total Student Attempts	Total Students Not Meeting Competency (%)	Total Track A Attempts	Track A (%) In-Person	Total Track B Attempts	Track B (%) Online	*p* Value *
PY1 Checking Station	40	7 (17.6)	25	6 (24)	15	1 (6.7)	0.16
PY1 MyDispense	26	4 (15.4)	17	3 (17.6)	9	1 (11.1)	0.66
PY1 Aliquot	66	14 (28)	34	6 (17.6)	32	2 (6.25)	0.16
PY1 Compounding	37	6 (16.2)	21	3 (14.3)	16	3 (18.8)	0.72
PY1 Geometric Dilution	25	3 (12)	13	3 (23.1)	12	0 (0)	0.08
PY2 Smoking Cessation	17	2 (11.8)	10	2 (20)	7	0 (0)	0.21
PY2 MyDispense	28	4 (14.3)	15	2 (13.3)	13	2 (15.4)	0.88
PY2 Compounding	68	1 (1.5)	38	0 (0)	30	1 (3.3)	0.26

* Chi-squared analysis.
